# Development and validation of a machine learning model for predicting venous thromboembolism complications following colorectal cancer surgery

**DOI:** 10.1186/s42492-025-00204-y

**Published:** 2025-09-12

**Authors:** Zongsheng Sun, Di Hao, Mingyu Yang, Wenzhi Wu, Hanhui Jing, Zhensong Yang, Anbang Sun, Wentao Xie, Longbo Zheng, Xixun Wang, Dongsheng Wang, Yun Lu, Guangye Tian, Shanglong Liu

**Affiliations:** 1 Department of Gastrointestinal Surgery, Affiliated Hospital of Qingdao University, Qingdao, 266000 Shandong China; 2 School of Control Science and Engineering, Shandong University, Jinan, 250100 Shandong China; 3 Department of Gastrointestinal Surgery, Yantai Yuhuangding Hospital, Yantai, 264000 Shandong China

**Keywords:** Colorectal cancer, Surgery, Complications, Venous thromboembolism, Machine learning, Prediction model

## Abstract

Postoperative venous thromboembolism (VTE) in colorectal cancer (CRC) patients undergoing surgery results in poor prognosis. However, there are no effective tools for early screening and predicting VTE. In this study, we developed a machine learning (ML)-based model for predicting the risk of VTE following CRC surgery and tested its performance using an external dataset. A total of 3227 CRC surgery patients were enrolled from the Affiliated Hospital of Qingdao University and Yantai Yuhuangding Hospital (from January 2016 to December 2023). Subsequently, 1596 patients from the Affiliated Hospital of Qingdao University were assigned to the training set, and 716 patients from Yantai Yuhuangding Hospital were assigned to the external validation set. A model was developed and trained using six ML algorithms using the stacking ensemble technique. Moreover, all models were developed using the tenfold cross-validation on the training set, and their performance was tested using an independent external validation set. In the training set, 173 (10.8%) patients developed VTE, 163 (10.2%) patients experienced deep venous thrombosis, and 29 (1.82%) cases had pulmonary embolism (PE). In the external validation set, 85 (11.9%) cases of VTE, 83 (11.6%) cases of deep vein thrombosis, and 14 (1.96%) cases of PE were recorded. The analysis revealed that the stacking model outperformed all other models in the external validation set, achieving significantly better performance in all metrics: the area under the receiver operating characteristic curve = 0.840 (0.790–0.887), accuracy = 0.810 (0.783–0.836), specificity = 0.819 (0.790–0.848), sensitivity = 0.741 (0.652–0.825), and recall = 0.959 (0.942–0.975). The stacking model for surgical CRC patients shows promise in enabling timely clinical detection of high-risk cases. This method facilitates the prioritized implementation of prophylactic anticoagulation in confirmed high-risk individuals, thereby mitigating unnecessary pharmacological intervention in low-risk populations.

## Introduction

Colorectal cancer (CRC) is the third most common cancer and the second leading cause of cancer-related death worldwide [1]. In China, most CRC patients are diagnosed when the disease is at advanced stages, when they are not suitable for surgical treatments, the primary treatment for CRC [2]. The surgical procedure for CRC is considered a challenging technique owing to the complex anatomical structure of the abdomen or pelvis, which can potentially damage non-surgical organs, resulting in postoperative complications [3]. Studies have shown that distant and even systemic complications frequently occur in CRC patients post-operation, owing to multiple factors. Postoperative venous thromboembolism (VTE) is one of the most common clinical complications [4, 5], with most cases occurring within 30 day after surgery. It is classified into deep vein thrombosis (DVT) and pulmonary embolism (PE) [6], with an estimated incidence of 1% to 20% [7, 8]. The risk of VTE in cancer patients is seven times greater than in the general population [9]. For example, hospitalized CRC patients have a higher risk and incidence of VTE owing to decreased postoperative activity and the systemic hypercoagulable state caused by the trauma, resulting in poor prognosis, prolonged bed rest, and an increased risk of organ embolism [10, 11]. The risk of VTE is even higher in elderly patients and those with thrombophilia. Notably, 5% to 10% of in-hospital deaths are directly caused by PE [12, 13]. 

Clinically established VTE risk assessment models (RAMs), such as the Caprini [14], Khorana [15], Padua [16], Wells score [17], and Rogers score [18], are valued for their simplicity and interpretability [19]. However, their dependence on a limited set of predefined risk factors reduces predictive accuracy and clinical applicability. Typically, significant variability exists in the application of the Caprini RAM across institutions, particularly regarding risk category definitions, cutoff thresholds, measurement protocols, and follow-up durations [14]. This inconsistency is highlighted by differing VTE incidence rates across cohorts within the same risk categories, reducing their clinical utility. The remaining representative traditional RAMs have some key limitations. Their risk stratification is limited by categorical classifications (e.g., low/medium/high risk), limiting its accuracy. In addition, these models rely on static frameworks with predetermined weights for risk factors (e.g., assigning 3 points for age ≥ 75 year) established through expert consensus. This fixed structure frequently results in risk underestimation, particularly in younger patients lacking traditional risk factors, and overlooks the integration of novel predictors or dynamic clinical parameters [20]. Moreover, conventional RAMs use linear weighting mechanisms that inadequately address non-linear interactions among risk factors [21]. To overcome the constraints of conventional models, we delved into machine learning (ML) techniques [22].

ML is a pivotal branch of artificial intelligence (AI) that allows computers to autonomously learn, infer, and make decisions by leveraging vast data sources [23, 24]. It is widely used in various fields, particularly in clinical medicine, where it allows for personalized diagnosis, treatment, and disease prediction through automated analysis and experiential learning. ML allows clinicians to optimize decision-making and increase overall work efficiency [25, 26]. A previous study by Brydges et al. [27] used ML to predict the occurrence of intestinal obstruction following colorectal surgery. They compared ML models’ accuracy to existing risk assessment tools. Another study showed that ML can accurately predict the risk of incision infection following a right hemicolectomy for colon cancer [28]. In the field of hematology, ML was used to predict the risk of bleeding in CAT patients undergoing anticoagulant therapy [29]. Several other investigations have shown that ML can identify high-risk groups of VTE in patients with acute diseases [30] or predict the chronic development of immune thrombocytopenia in children [31]. Numerous studies have shown that ML can integrate diverse risk factors in CRC patients, such as genetic mutations, colonoscopic images, fecal occult blood tests, and blood biomarkers [22, 32]. ML efficiently processes heterogeneous data from genomes, images, and pathology reports to extract advanced features, addressing the inherent heterogeneity in CRC patients. However, the variety of ML algorithms poses a challenge.

In this study, we developed some ML models based on preoperative and intraoperative indicators to predict the occurrence of VTE in CRC patients undergoing radical resection. The results of this study are expected to allow clinicians to make timely adjustments to the diagnosis and treatment plan, thereby reducing the risk of complications.

## Methods

### Study design and population

**Fig. 1:**
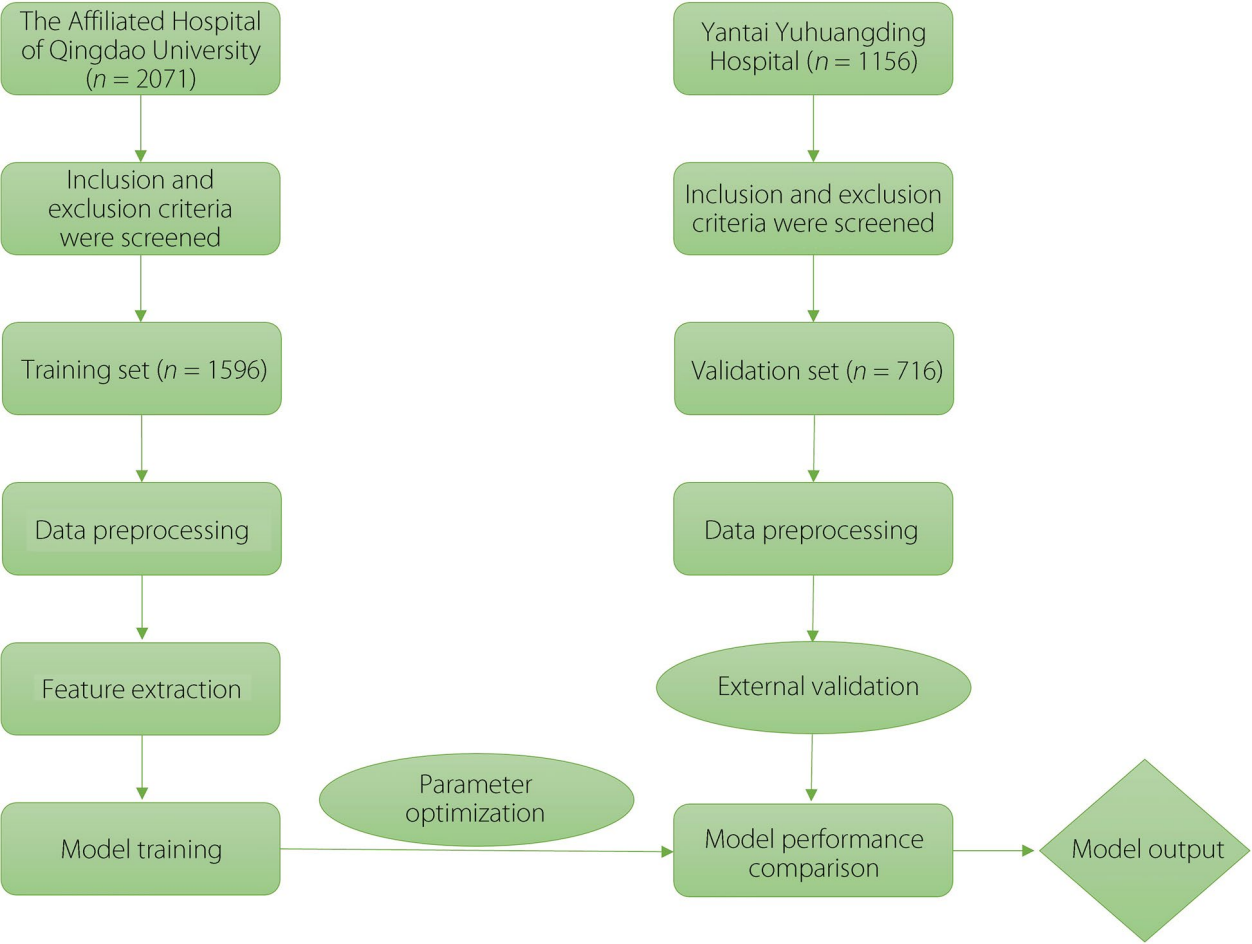
Overall study protocol and design of the study

The study was approved by the Ethics Committee of the Affiliated Hospital of Qingdao University (ethics No. QYFY WZLL 29437). Because this was a multicenter retrospective study, informed consent was not required. Figure 1 shows the overall study design and analytical procedure. A total of 3227 patients diagnosed with CRC based on ICD-9-CM guidelines and underwent surgery at the Affiliated Hospital of Qingdao University and Yantai Yuhuangding Hospital from January 2016 to December 2023 were consecutively enrolled in the study. All surgical procedures and perioperative care were performed by a team of gastrointestinal surgeons. Inclusion criteria were: (1) age over 18 years old; (2) colorectal malignant tumor diagnosed by histopathology and radical resection; (3) preoperative chest computed tomography (CT) and abdominal CT were performed to confirm the absence of distant metastasis; (4) stay in the hospital for at least 7 day; (5) no VTE or anticoagulant therapy within 90 day before surgery; (6) VTE confirmed by vascular ultrasound within 30 day after surgery. Exclusion criteria included: (1) emergency surgery because of perforation and bleeding; (2) the presence of distant metastasis; (3) patients with VTE or receiving anticoagulant therapy within 90 day before surgery; (4) preoperative neoadjuvant chemoradiotherapy; (5) N-thrombotic death during hospitalization or less than 30 day of follow-up; and (6) coagulation function and other related laboratory tests were not definitive, or significant clinical data were lacking.

### Data collection and preprocessing

Clinical data and perioperative examination information for the patients were collected retrospectively. This included imaging results, laboratory indicators, and risk scores. For patients hospitalized for more than 7 day but fewer than 30 day, two investigators thoroughly reviewed their medical records and Made telephone follow-ups to contact either the patient or their family for additional information. This method was used to confirm whether any new-onset VTE occurred within 30 day of surgery, with subsequent verification from another institution after discharge. Once the relevant data were collected, it was preprocessed to encode the attributes of patient variables using label encoding for ordinal categorical variables and one-hot encoding for unordered categorical variables. Subsequently, outlier detection was performed. For continuous variables, Z-score statistics were used to identify abnormal data, which were set as Not a Number. Following the removal of outliers, missing values were imputed using multiple imputations based on Bayesian ridge regression for missing values for continuous variables and random imputation for categorical variables.

### Outcome and variable selection

The primary outcome was a newly diagnosed non-central venous catheter VTE, including DVT and PE, within 30 day of surgery. VTE can be diagnosed using vascular ultrasound imaging and chest CT imaging. Briefly, patients who were bedridden or immobilized underwent a venous bedside ultrasound of their lower extremities. The occurrence of PE was confirmed using pulmonary artery CT angiography. Several clinical symptoms associated with VTE were collected, including limb edema, unexplained pain, fever, shortness of breath, gait disorder, and abnormal laboratory tests. In addition, the Lasso regression and grid search were used to select the best-regularized strength coefficient and ensure that the feature weight matrix was sparse. The features with non-zero weight were collected, and a regularized coefficient screening map was created.

### Stacking framework

The stacking ensemble architecture generates meta-features from base model probabilistic predictions, which are then integrated as input vectors for the stacking model. To avoid data leakage and ensure robust generalization, we used a nested stratified tenfold cross-validation scheme, with primary folds preserving class distribution and secondary folds facilitating hyperparameter tuning within each training partition. In each cross-validation cycle, base models were fitted to the training subset (9 folds), and the validation fold generated class probability estimates, filling designated columns in the meta-feature matrix. The complete meta-feature tensor was constructed by horizontally concatenating all cross-validated predictions, resulting in an optimized input space for the stacking model’s final training phase. During base model training, ML algorithms differ in assumptions about data distributions (linear or nonlinear), noise tolerance, and feature space characteristics (continuous or discrete), necessitating careful alignment with dataset characteristics. High-bias models, such as logistic regression (LG), generalize well but frequently underfit complex patterns, while high-variance architectures, such as multilayer perceptron (MLP), perform well with large datasets but risk overfitting. To balance the bias-variance trade-offs while maintaining computational efficiency and interpretability, we prioritized diversity among base models, selecting complementary methods. Base models underwent systematic hyperparameter optimization using grid search, Bayesian optimization, and randomized sampling.

### Model construction and evaluation

The training set consisted of patients from the Affiliated Hospital of Qingdao University, while those from Yantai Yuhuangding Hospital were assigned to the external validation set. Six ML algorithms, including LG, random forest (RF), support vector machine (SVM), MLP, eXtreme gradient boosting (XGB), and CatBoost, were used to build and optimize models in the training set. In addition, the stacking ensemble concept was used to train the stacking model to achieve the optimal integration of all model results. To improve model interpretability, Shapley additive explanations (SHAP) was used to assess each feature’s positive or negative influence on VTE prediction, while a feature importance map was generated to visualize the contribution of individual variables. Model performance was comprehensively evaluated using receiver operating characteristic (ROC) curves and area under the curve (AUC) calculations, as well as key metrics such as accuracy, specificity, sensitivity, and recall, all reported with their respective 95%CIs for objective comparison. An external validation set was used to further verify the model’s robustness.

### Statistical analysis

Continuous variables were analyzed using the student’s *t*-test and Mann-Whitney sum test, while categorical variables were analyzed using the chi-square or Fisher’s exact test. All statistical tests were two-sided, and *P* < 0.05 was considered statistically significant. R-4.3.2 software was used to conduct statistical analyses and build models.

## Results

### Baseline characteristics

A total of 1596 CRC patients who underwent radical resection in the Affiliated Hospital of Qingdao University were assigned to the training set, and 716 CRC patients who underwent radical resection of CRC in Yantai Yuhuangding Hospital were assigned to the external test set. In the training set, 48.1% of non-VTE patients were ≥ 65 year, 63.9% were Male, 60.6% underwent rectectomy, and 39.4% underwent colon resection. Moreover, 59.5% of VTE patients were ≥ 65 year, 63.0% were Male, 37.0% underwent rectectomy, and 63.0% underwent colon resection. In the external validation set, 48.8% of non-VTE patients were ≥ 65 year, 67.4% were Male, 60.1% underwent rectectomy, and 39.9% underwent colon resection. Moreover, 58.8% of VTE patients were ≥ 65 year, 65.9% were Male, 40.0% underwent rectectomy, and 60.0% underwent colon resection. The incidence of VTE in the two cohorts was 173 (10.8%) and 85 (11.9%). In the training cohort, there were 163 (10.2%) DVT patients and 29 (1.82%) PE patients, while the external validation cohort had 83 (11.6%) DVT patients and 14 (1.96%) PE patients. Further analysis identified significant differences in length of stay, white blood cell (WBC) count, hemoglobin (Hb), activated partial thromboplastin time (APTT), alanine aminotransferase (ALT), American Society of Anesthesiologists Physical Status Classification (ASA), myoglobin (Mb), antithrombin III (AT-III), D-dimer, prealbumin (PA), and surgical site between non-VTE patients and VTE patients in both centers (*P* < 0.05) (Table 1).

**Table 1:** Baseline data characteristics of the training and validation sets

Variable	Training set (*n* = 1596)					Validation set (*n* = 716)			
	non-VTE *n* = 1423			VTE *n* = 173	*P*	non-VTE *n* = 631		VTE *n* = 85	*P*
Age, *n* (%)					0.006				0.106
< 65	738 (51.9)		70 (40.5)			323 (51.2)		35 (41.2)	
≥ 65	685 (48.1)		103 (59.5)			308 (48.8)		50 (58.8)	
Gender, *n* (%)					0.873				0.882
Female	513 (36.1)		64 (37.0)			206 (32.6)		29 (34.1)	
Male	910 (63.9)		109 (63.0)			425 (67.4)		56 (65.9)	
BMI, mean (SD)	24.31 (3.35)		24.59 (4.14)		0.324	23.85 (3.72)		23.96 (4.54)	0.810
Length of stay (d), mean (SD)	14.86 (8.12)		20.89 (10.42)		< 0.001	19.97 (12.16)		20.39 (11.55)	0.035
WBC (10^9/L), mean (SD)	6.03 (2.63)		6.84 (2.47)		< 0.001	6.79 (4.39)		8.69 (8.23)	0.001
Hb (g/L), mean (SD)	128.48 (22.89)		118.80 (27.21)		< 0.001	127.11 (22.25)		121.60 (23.92)	0.034
Plt (10^9/L), mean (SD)	243.34 (75.79)		274.60 (102.18)		< 0.001	236.68 (77.66)		235.51 (90.20)	0.898
PT (s), mean (SD)	10.28 (4.37)		10.04 (11.82)		0.597	11.50 (4.40)		11.99 (2.91)	0.321
APTT (s), mean (SD)	27.85 (11.13)		23.03 (17.21)		< 0.001	27.13 (12.45)		24.36 (19.64)	< 0.001
AT III (%), mean (SD)	96.37 (16.37)		88.03 (19.65)		< 0.001	91.21 (18.43)		82.90 (23.56)	< 0.001
ALT (U/L), mean (SD)	18.03 (14.47)		15.64 (9.74)		0.035	20.83 (21.60)		26.70 (40.44)	0.039
AST (U/L), mean (SD)	19.89 (21.63)		18.98 (10.57)		0.585	21.16 (21.52)		26.69 (32.10)	0.038
Albumin (g/L), mean (SD)	44.37 (10.50)		38.26 (6.55)		0.064	43.44 (7.62)		34.84 (9.03)	< 0.001
D-dimer (ng/mL), mean (SD)	337.98 (341.28)		1117.26 (2655.70)		< 0.001	542.28 (704.99)		1990.14 (2374.32)	< 0.001
FIB (g/L), mean (SD)	3.32 (1.07)		3.84 (3.26)		< 0.001	3.47 (2.55)		3.46 (1.46)	0.967
Cr (μmol/L), mean (SD)	79.65 (37.74)		76.73 (24.51)		0.321	84.51 (57.25)		82.72 (41.26)	0.780
UA (μmol/L), mean (SD)	317.12 (84.80)		337.56 (101.93)		0.077	319.06 (93.27)		321.34 (88.69)	0.832
TG (mmol/L), mean (SD)	1.36 (2.84)		1.23 (0.73)		0.563	1.29 (0.74)		1.23 (0.89)	0.485
TC (mmol/L), mean (SD)	4.86 (1.20)		4.73 (1.16)		0.094	4.65 (1.31)		4.32 (1.47)	0.029
LDL (mmol/L), mean (SD)	3.01 (0.83)		3.16 (3.28)		0.140	2.83 (0.98)		2.67 (0.98)	0.159
K (mmol/L), mean (SD)	4.14 (0.43)		4.14 (0.53)		0.838	4.12 (0.49)		4.14 (0.56)	0.823
FBG (mmol/L), mean (SD)	5.66 (2.46)		5.49 (3.95)		< 0.001	5.52 (6.14)		5.89 (2.92)	0.579
Ca (mmol/L), mean (SD)	2.28 (0.45)		2.29 (0.64)		0.759	2.31 (0.83)		2.12 (0.60)	0.044
CKMB (ng/mL), mean (SD)	1.41 (2.95)		1.40 (2.08)		0.974	1.65 (2.36)		2.06 (2.48)	0.141
Mb (ng/mL), mean (SD)	35.64 (24.02)		43.62 (38.98)		< 0.001	46.09 (62.25)		53.73 (68.52)	0.001
PA (mg/L), mean (SD)	232.66 (91.16)		184.43 (79.08)		< 0.001	223.72 (82.46)		182.04 (87.96)	< 0.001
DBIL (μmol/L), mean (SD)	4.22 (2.30)		4.25 (2.32)		0.877	5.04 (3.48)		4.60 (3.86)	< 0.001
IBIL(μmol/L), mean (SD)	10.27 (5.66)		9.28 (5.24)		0.030	10.82 (6.71)		11.30 (8.65)	0.548
ASA, *n* (%)					< 0.001				0.005
< 3	1012 (71.1)		45 (26.0)			505 (80.0)		56 (65.9)	
≥ 3	411 (28.9)		128 (74.0)			126 (20.0)		29 (34.1)	
NRS-2002, *n* (%)					0.070				0.784
< 3	267 (18.8)		43 (24.9)			268 (42.5)		38 (44.7)	
≥ 3	1156 (81.2)		130 (75.1)			363 (57.5)		47 (55.3)	
Caprini score, *n* (%)					< 0.001				0.476
< 5	1392 (97.8)		138 (79.8)			549 (87.0)		71 (83.5)	
≥ 5	31 (2.2)		35 (20.2)			82 (13.0)		14 (16.5)	
T stage, *n* (%)					0.007				0.171
T1	102 (7.1)		15 (8.7)			37 (5.9)		2 (2.4)	
T2	226 (15.9)		20 (11.6)			64 (10.1)		11 (12.9)	
T3	636 (44.7)		99 (57.2)			299 (47.4)		48 (56.5)	
T4	459 (32.3)		39 (22.5)			231 (36.6)		24 (28.2)	
N stage, *n* (%)					0.538				0.374
N0	456 (32.0)		59 (34.1)			226 (35.8)		33 (38.8)	
N1	502 (35.3)		62 (35.8)			218 (34.5)		27 (31.8)	
N2	465 (32.7)		52 (30.1)			187(29.7)		25 (29.4)	
Differentiation, *n* (%)					0.927				0.137
Low	465 (32.7)		54 (31.2)			196 (31.1)		30 (35.3)	
Moderate	699 (49.1)		87 (50.3)			328 (51.9)		35 (41.2)	
High	259 (18.2)		32 (18.5)			107 (17.0)		20 (23.5)	
Operation, *n* (%)					< 0.001				0.905
Laparoscopy	1077 (75.7)		86 (49.7)			483 (76.5)		64 (75.3)	
Open	346 (24.3)		87 (50.3)			148 (23.5)		21 (24.7)	
Surgical site, *n* (%)					< 0.001				0.001
Colon	560 (39.4)		109 (63.0)			252 (39.9)		51 (60.0)	
Rectum	863 (60.6)		64 (37.0)			379 (60.1)		34 (40.0)	
DM, *n* (%)					0.023				0.811
No	1231 (86.5)		138 (79.8)			539 (85.4)		74 (87.1)	
Yes	192 (13.5)		35 (20.2)			92 (14.6)		11 (12.9)	
HP, *n* (%)					0.004				0.178
No	1034 (72.7)		107 (61.8)			491 (77.8)		60 (70.6)	
Yes	389 (27.3)		66 (38.2)			140 (22.2)		25 (29.4)	
CHD, *n* (%)					0.001				0.603
No	1330 (93.5)		149 (86.1)			579 (91.8)		76 (89.4)	
Yes	93 (6.5)		24 (13.9)			52 (8.2)		9 (10.6)	
ICH, *n* (%)					1.000				0.621
No	1414 (99.4)		172 (99.4)			623 (98.7)		85 (100.0)	
Yes	9 (0.6)		1 (0.6)			8 (1.3)		0 (0.0)	
CVT, *n* (%)					0.159				0.246
No	1394 (98.0)		166 (96.0)			613 (97.1)		80 (94.1)	
Yes	29 (2.0)		7 (4.0)			18 (2.9)		5 (5.9)	
PUI, *n* (%)					0.003				0.640
No	1361 (95.6)		156 (90.2)			266 (42.2)		33 (38.8)	
Yes	62 (4.4)		17 (9.8)			365 (57.8)		52 (61.2)	
Smoke, *n* (%)					0.731				0.669
No	1014 (71.3)		126 (72.8)			404 (64.0)		57 (67.1)	
Yes	409 (28.7)		47 (27.2)			227 (36.0)		28 (32.9)	
Drink, *n* (%)					0.990				0.878
No	1097 (77.1)		134 (77.5)			466 (73.9)		64 (75.3)	
Yes	326 (22.9)		39 (22.5)			165 (26.1)		21 (24.7)	
Postoperative complication									
Death, *n* (%)					0.086				1.000
No	1421 (99.9)		171 (98.8)			629 (99.7)		85 (100.0)	
Yes	2 (0.1)		2 (1.2)			2 (0.3)		0 (0.0)	
Po-CVT, *n* (%)					0.519				0.537
No	1422 (99.9)		172 (99.4)			626(99.2)		83 (97.6)	
Yes	1 (0.1)		1 (0.6)			5(0.8)		2 (2.4)	
POP, *n* (%)					0.854				0.855
No	1382 (97.1)		169 (97.7)			622 (98.6)		83 (97.6)	
Yes	41 (2.9)		4 (2.3)			9 (1.4)		2 (2.4)	
AL, *n* (%)					0.011				0.493
No	1365 (95.9)		158 (91.3)			551 (87.3)		77 (90.6)	
Yes	58 (4.1)		15 (8.7)			80 (12.7)		8 (9.4)	
SSI, *n* (%)					< 0.001				0.805
No	1359 (95.5)		152 (87.9)			516 (81.8)		68 (80.0)	
Yes	64 (4.5)		21 (12.1)			115 (18.2)		17 (20.0)	
IAI, *n* (%)					< 0.001				0.680
No	1394 (98.0)		159 (91.9)			550 (87.2)		76 (89.4)	
Yes	29 (2.0)		14 (8.1)			81 (12.8)		9 (10.6)	
Po-bleeding*, n (%)*					0.004				0.855
No	1386 (97.4)		161 (93.1)			594 (94.1)		81 (95.3)	
Yes	37 (2.6)		12 (6.9)			37 (5.9)		4 (4.7)	
DVT, *n* (%)					< 0.001				< 0.001
No	1423 (100.0)		10 (5.8)			631 (100.0)		2 (2.4)	
Yes	0 (0.0)		163 (94.2)			0 (0.0)		83 (97.6)	
PE, *n* (%)					< 0.001				< 0.001
No	1423 (100.0)		144 (83.2)			631 (100.0)		71 (83.5)	
Yes	0 (0.0)		29 (16.8)			0 (0.0)		14 (16.5)	

*BMI* Body mass index, *Plt* Platelet count, *PT* Prothrombin time, *AST* Aspartate aminotransferase, *FIB* Fibrinogen, *Cr* Serum creatinine, *UA* Uric acid, *TG* Triglyceride, *TC* Total cholesterol, *LDL* Low-density lipoprotein, *FBG* Fasting blood glucose value, *CKMB* Creatine kinase isoenzymes, *DBIL* Direct bilirubin, *IBIL* Indirect bilirubin, *DM* Diabetes mellitus, *HP* High blood pressure, *CHD* Coronary heart disease, *ICH* Intracerebral hemorrhage, *CVT* Cerebral thrombus, *PUI* Preoperative pneumonia, *po-CVT* Postoperative-CVT, *POP* Postoperative pneumonia, *AL* Anastomotic leak, *SSI* Surgical site infection, *IAI* Intra-abdominal infection, *Po-bleeding* Postoperative-bleeding

### Feature selection

**Fig. 2:**
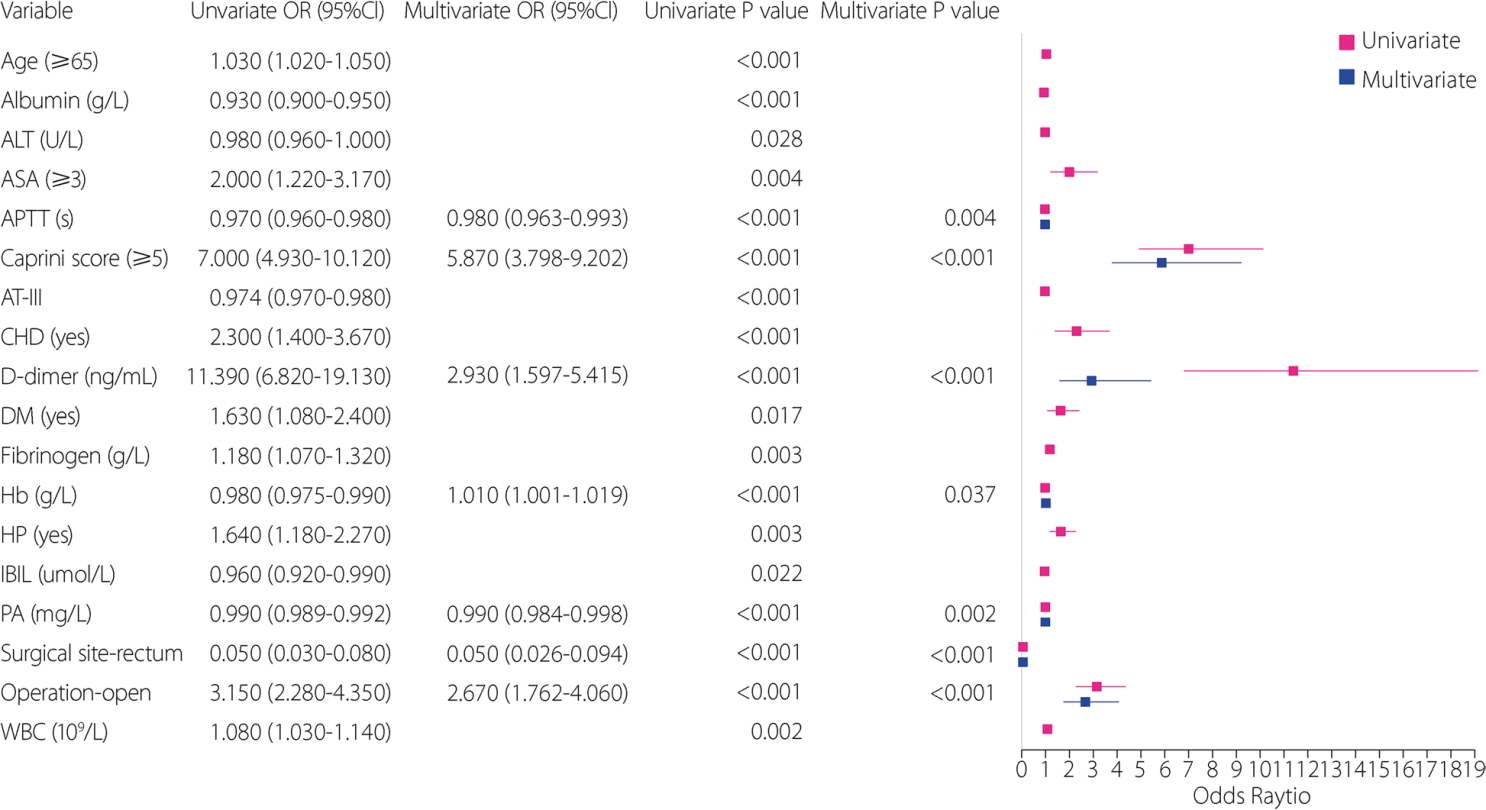
Univariate and multivariate LG analyses. Variables with *P* < 0.05 in the univariate analysis are included in the multivariate analysis. Among the variables, APTT, Caprini score, D-dimer, Hb, PA, surgical site, and operation remained statistically significant in the multivariate analysis (*P* < 0.05). High Caprini score, high level of D-dimer, and open surgery are significant independent risk factors for outcome

**Fig. 3:**
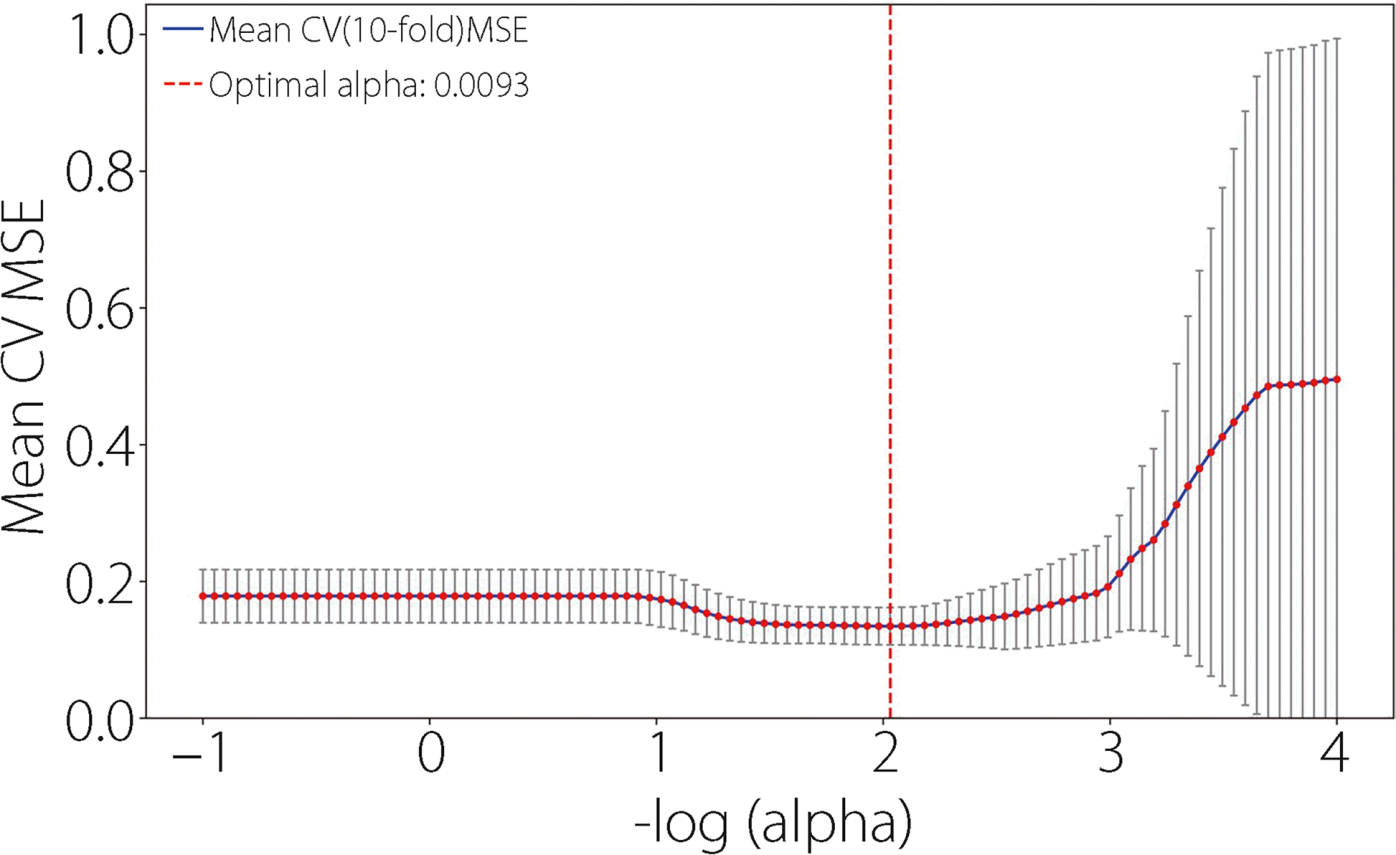
Ten-fold cross‐validation for optimal regularization strength coefficient selection in the Lasso regression. The U-shaped curve illustrates the process in which as alpha increases, the model becomes simpler, and the cross-validation error first drops to the optimal equilibrium point and then rises (the model underfits). The alpha value at the bottom of “U” represents the minimum prediction error on the cross-validation set. At this juncture, the model retains only essential features, ensuring prediction accuracy while mitigating overfitting risks

**Fig. 4:**
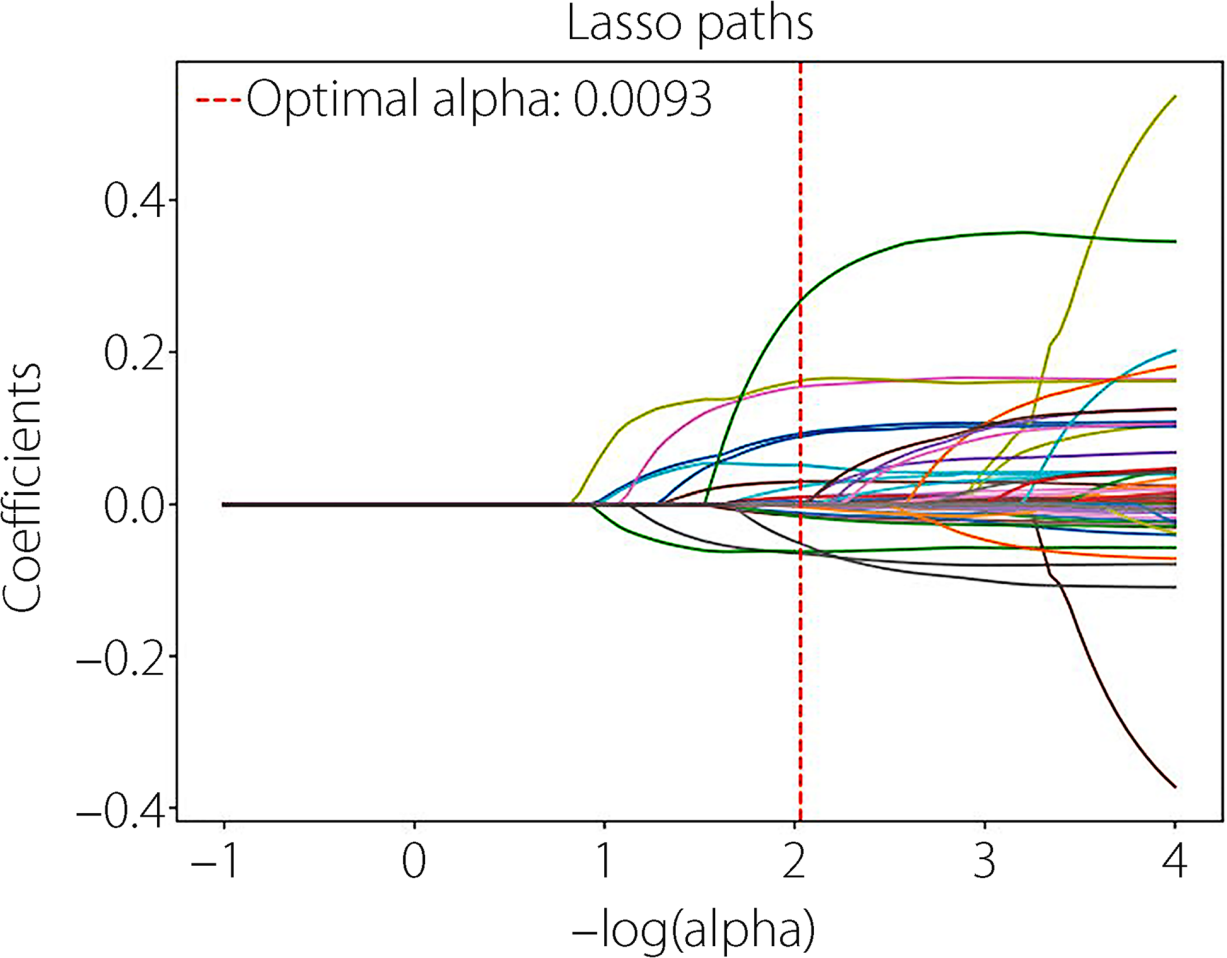
The effects of different regularization parameters (alpha) on regression coefficients in Lasso regression: the horizontal axis indicates the log value of the regularization parameter, and from left to right, the regularization is gradually increased. The vertical axis denotes the value of the regression coefficient. Each curve corresponds to the coefficient change trajectory of a feature

To compare traditional models and ML in feature selection, we initially conducted conventional LG feature analysis. Univariate and multivariate analyses identified variables with *P* value below 0.05, including surgical site, Caprini score, D-dimer, operation, APTT, PA, and Hb (Fig. 2). Following the LG multifactorial results, Lasso regression was used for feature selection to train ML models. The optimal regularization strength coefficient was determined using Lasso regression and grid search. To further analyze the impact of feature importance and regularization strength on model sparsity, we plotted the Lasso ten-fold cross-validation diagram (Fig. 3) helping us select the optimal alpha that achieves a balance between sufficient sparsity and predictive accuracy, and plotted the Lasso path diagram (Fig. 4) showing the dynamic changes of each feature weight as the regularization coefficient changes. At the optimal regularization strength coefficient (alpha = 0.0093), 15 features with Weight not 0 were obtained: surgical site, Caprini score, D-dimer, operation, length of stay, LDL, APTT, AT-III, NRS-2002, differentiation, albumin, Hb, Ca, TBIL, and Plt. At this point, the model contained the fewest necessary features, effectively addressing feature redundancy while ensuring optimal model performance.

### Feature analysis and model interpretation

**Fig. 5:**
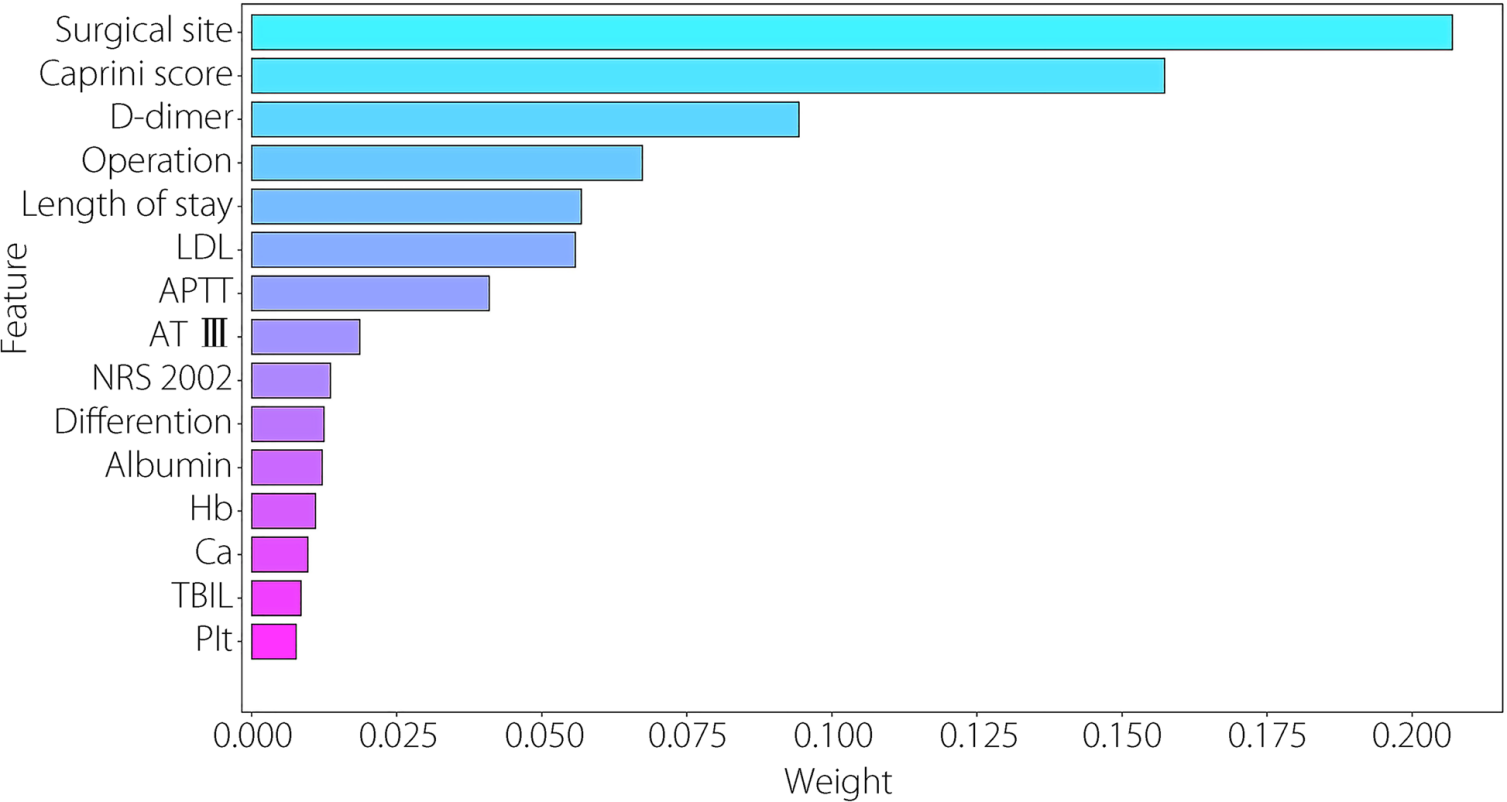
Feature importance strip diagram: after feature selection, the importance of the 15 features in the model is determined based on the weight, and the importance order decreases from top to bottom. The ordinate represents the feature name and the ordinate denotes the weight value

**Fig. 6:**
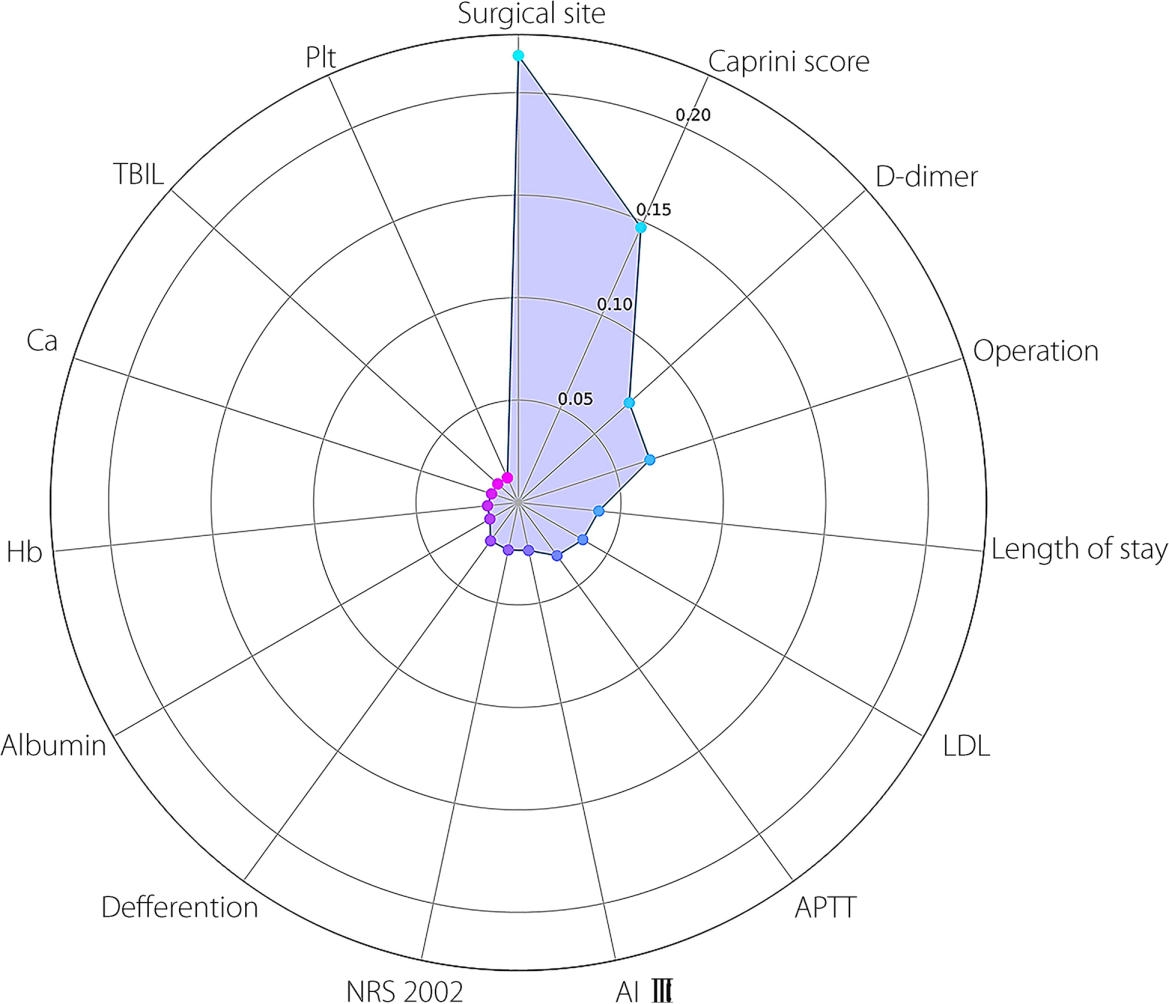
Radar map: Each axis represents an analytical dimension (a feature), and each dimension extends outward from a central point to form a polygon. The closer to the center, the lower the value, and the closer to the edge, the higher the value

**Fig. 7:**
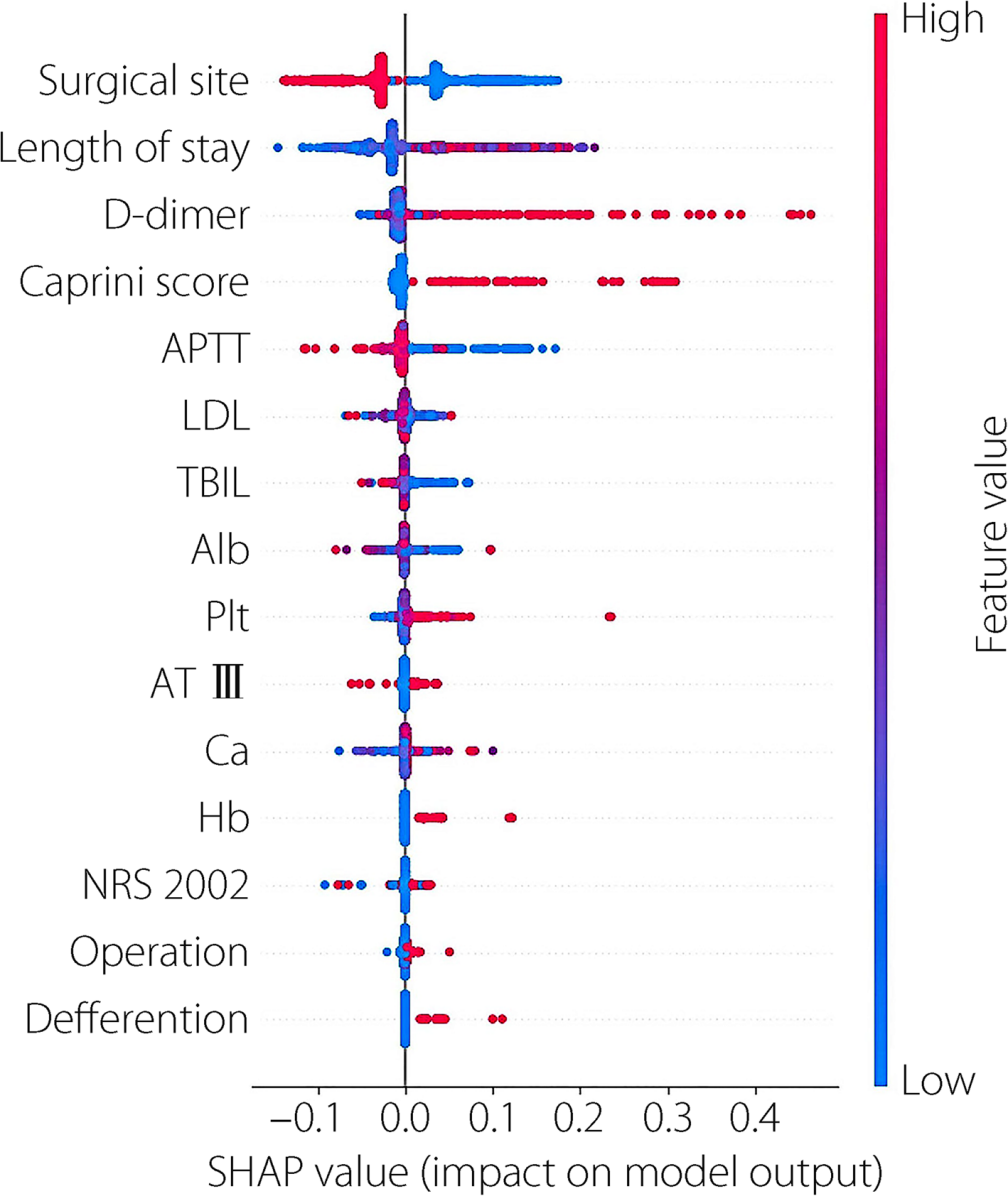
The horizontal coordinate indicates the size of the SHAP value, showing the degree of influence of features on outcome prediction. The ordinate is the feature name. If red is mainly in positive coordinates, the higher the eigenvalue, the more positive the contribution to the prediction is. If blue is mainly in the negative coordinates, the lower the eigenvalue, the more outstanding the negative contribution to the prediction. If red corresponds to a negative SHAP value, the higher the feature value, the lower the outcome risk

To further explain the role of each feature in the model and better understand the model’s decision-making process, we analyzed the importance of the selected features and compared them through weight ranking (Fig. 5). The five most significant features, ranked from highest to lowest, were surgical site, Caprini score, D-dimer, operation, and length of stay. A radar chart was then drawn to show each feature’s impact on the model’s comprehensive performance (Fig. 6). The SHAP diagram of the stacking model showed that rectal resection, open surgery, high D-dimer, high Caprini score, and short APTT positively predicted VTE in patients (Fig. 7).

### Model performance comparisons

**Fig. 8:**
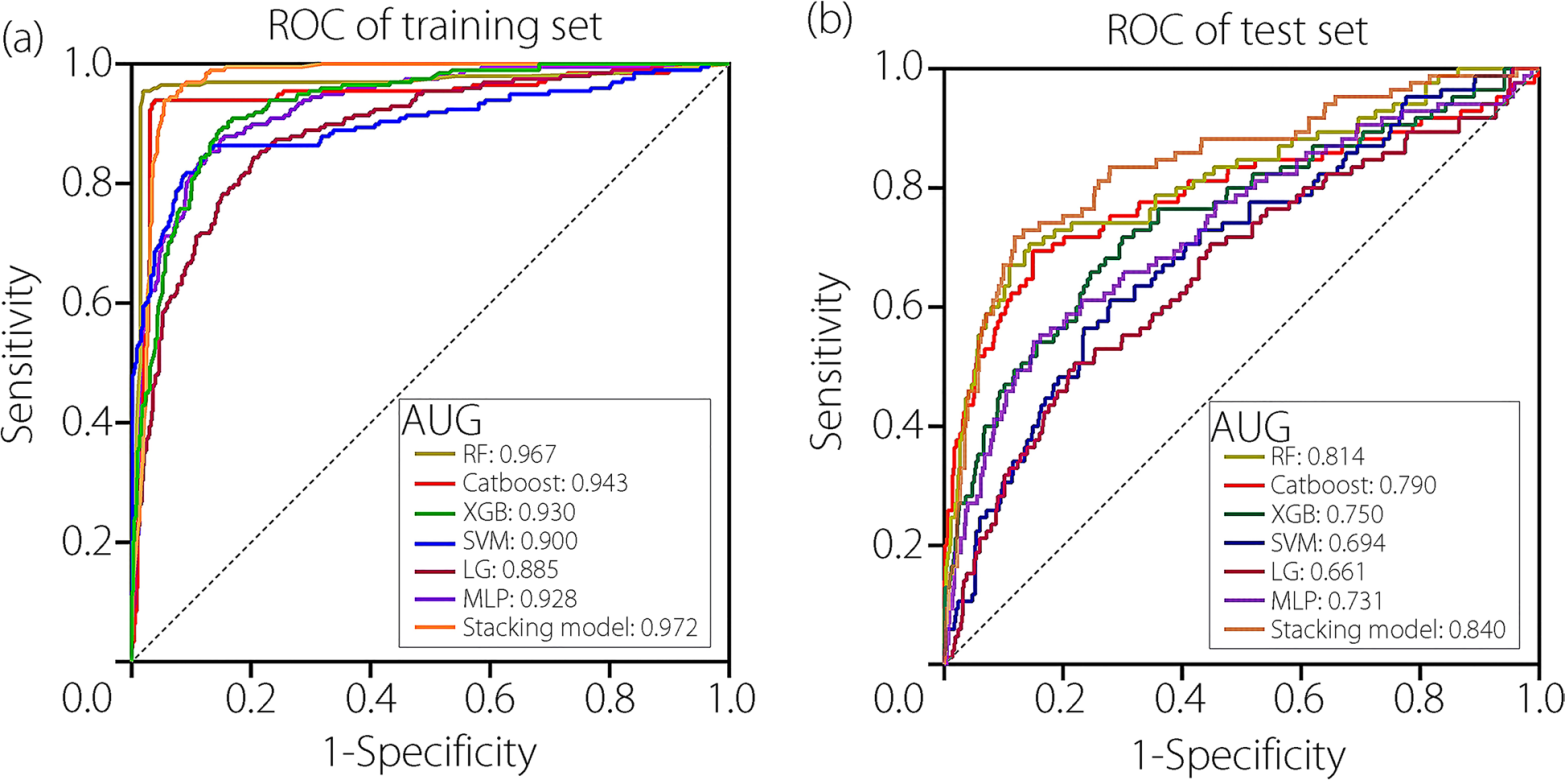
Comparison of model performance in predicting postoperative VTE using different modeling approaches. **a** Prediction results for all models in the training set; **b** Prediction results for all models in the external validation set. The figure demonstrates that stacking model achieved the best performance: train-AUC = 0.972 (0.962–0.981); validation-AUC = 0.840 (0.790–0.887)

The optimal prediction results from tenfold cross-validation on the training set revealed that the stacking model had the highest performance, with an AUC of 0.972 (0.962–0.981), accuracy of 0.940 (0.924–0.955), specificity of 0.951 (0.934–0.966), sensitivity of 0.904 (0.856–0.944), and recall of 0.971 (0.957–0.984). In addition to the stacking model, RF showed good prediction performance, with an AUC value of 0.967 (Table 2, Fig. 8). External validation results showed that the stacking model still had the highest prediction efficiency among all models in different cohorts, with an AUC of 0.840 (0.790–0.887), accuracy of 0.810 (0.783–0.836), specificity of 0.819 (0.790–0.848), sensitivity of 0.741 (0.652–0.825), and recall of 0.959 (0.942–0.975). By examining variations in each index across different datasets, we discovered that the stacking model showed superior stability compared to other models, highlighting its robustness and reliability in predictive tasks across diverse environments.

**Table 2:** The predicted results of all models in the training set and the validation set

Model	Data set	AUC (95%CI)	Accuracy (95%CI)	Specificity (95%CI)	Sensitivity (95%CI)	Recall (95%CI)
LG	Training	0.885 (0.856–0.913)	0.857 (0.832–0.879)	0.950 (0.933–0.966)	0.540 (0.471–0.612)	0.875 (0.851–0.898)
	Validation	0.661 (0.594–0.723)	0.778 (0.744–0.807)	0.824 (0.792–0.855)	0.435 (0.329–0.538)	0.916 (0.892–0.936)
RF	Training	0.967 (0.945–0.984)	0.975 (0.964–0.984)	0.981 (0.970–0.990)	0.955 (0.922–0.981)	0.987 (0.977–0.994)
	Validation	0.814 (0.758–0.869)	0.823 (0.795–0.852)	0.838 (0.808–0.868)	0.706 (0.608–0.798)	0.955 (0.936–0.972)
SVM	Training	0.900 (0.867–0.929)	0.888 (0.867–0.909)	0.969 (0.954–0.981)	0.611 (0.544–0.680)	0.894 (0.872–0.917)
	Validation	0.694 (0.630–0.753)	0.803 (0.774–0.831)	0.865 (0.838–0.891)	0.341 (0.238–0.438)	0.907 (0.884–0.929)
MLP	Training	0.928 (0.873–0.914)	0.893 (0.933–0.965)	0.950 (0.638–0.769)	0.702 (0.638–0.769)	0.916 (0.894–0.936)
	Validation	0.731 (0.668–0.789)	0.768 (0.736–0.800)	0.794 (0.764–0.826)	0.577 (0.464–0.679)	0.933 (0.911–0.953)
XGB	Training	0.930 (0.911–0.947)	0.878 (0.855–0.899)	0.944 (0.926–0.960)	0.657 (0.593–0.718)	0.903 (0.879–0.924)
	Validation	0.750 (0.690–0.809)	0.828 (0.802–0.856)	0.873 (0.847–0.898)	0.494 (0.395–0.600)	0.928 (0.907–0.947)
CatBoost	Training	0.943 (0.918–0.965)	0.958 (0.944–0.971)	0.969 (0.955–0.982)	0.919 (0.880–0.952)	0.976 (0.964–0.986)
	Validation	0.790 (0.728–0.853)	0.772 (0.742–0.806)	0.780 (0.746–0.815)	0.718 (0.625–0.813)	0.954 (0.935–0.971)
Stacking	Training	0.972 (0.962–0.981)	0.940 (0.924–0.955)	0.951 (0.934–0.966)	0.904 (0.856–0.944)	0.971 (0.957–0.984)
	Validation	0.840 (0.790–0.887)	0.810 (0.783–0.836)	0.819 (0.790–0.848)	0.741 (0.652–0.825)	0.959 (0.942–0.975)

## Discussion

Data on CRC patients were obtained from two centers retrospectively. Seven ML algorithms were used to develop models for predicting VTE after CRC surgery, which were then externally validated using an independent dataset. To better integrate the prediction performance of each model and maximize benefits, the stacking model was trained using the stacking ensemble concept. The models were also compared to other robust classifier ML models, revealing that the stacking model had the best prediction performance, followed by RF, and LG exhibited the worst performance. The results showed that the stacking model developed in this study is expected to improve early diagnosis and treatment of VTE, reducing the risk of poor prognosis and mortality. In addition, it will enable clinicians to identify and explain preoperative risk factors associated with VTE, thereby making evidence-based and scientifically sound clinical decisions.

Stacking is an ensemble learning method also known as stacked generalization. The stacking ensemble learning framework uses a stacking model (meta-model or meta-classifier), to integrate predictions from six diverse base algorithms. By leveraging the algorithms’ complementary strengths, the stacking model synthesizes insights from various data distribution assumptions, reducing systematic bias and increasing robustness [33–35]. In contrast to complex models prone to overfitting with limited samples, this method uses cross-validated out-of-fold predictions from base model validation outputs to ensure generalizability before training the stacking model. It dynamically optimizes the combination of base model outputs, such as probability estimates from LG or class probabilities from RFs, using adaptive weight allocation, error correction, and high-order feature extraction. Specifically, it assigns greater weights to base models that perform well in specific sample subgroups and resolves prediction conflicts caused by divergent errors (e.g., MLP overestimating risk *vs* LG underestimating risk) using statistical error pattern analysis [36]. By treating base model predictions as high-order abstractions of raw features, the stacking model captures complex interaction patterns beyond the reach of individual algorithms. In addition, it improves data adaptability by addressing the challenge of limited VTE positive samples using the built-in weighting strategy of XGB/CatBoost, managing imbalanced datasets, and further calibrating probability outputs [35, 37].

Traditional RAMs necessitate scoring systems designed for specific populations. For example, Caprini’s validation, which is based on historical cohorts, lacks generalizability across populations and is predominantly used in surgical settings, while Padua is primarily used in internal medicine [22]. Originating from European and American populations, these models exhibit inadequate calibration for Asian populations [23]. This geographical incongruity highlights the importance of models capable of adapting dynamically to regional epidemiological characteristics. Stacking addresses model mismatch stemming from regional and population variations by providing incremental updates and dynamic weight adjustments. This technique allows for quick weight adjustments through online learning without necessitating re-training on the entire dataset. Moreover, stacking facilitates regional generalization by segmenting the validation set based on geographical regions or populations during training, compelling the model to develop cross-region generalization capabilities. Adversarial training can be incorporated to improve the stacking model by encouraging it to disregard non-causal distinctions and focus solely on epidemiological variances genuinely linked to VTE.

Common statistical methods, such as linear regression and LG, focus on the relationship between explanatory variables and results, allowing for model interpretation based on the distribution state of data. However, this method has limited processing capacity when handling high-dimensional data or complex data structures [36, 38]. In contrast, the ML technique does not rely on distribution assumptions and can learn complex patterns and features from data, making it more suitable for processing large-scale, high-dimensional, multimodal data, such as images, text, audio, etc. For example, strong classifiers (RF, XGB, MLP) can mine patterns from data using automated feature extraction, nonlinear modeling, and flexible parameter adjustment, producing better results in diverse practical settings. Moreover, traditional statistical analyses are difficult to perform because they require significant computational resources or model assumptions failure [39–42]. However, the ML technique focuses on the predictive power of new data rather than simply explaining the relationship between variables. It also supports upgrading and optimization. For example, methods such as RF and XGB significantly improve the model’s robustness and predictive performance by integrating multiple weak classifiers into strong classifiers [43].

Conventional ensemble methods such as bagging and boosting in ML face limitations in base model diversity. Bagging uses self-sampling to generate multiple sub-training sets for parallel training of homogeneous base models, primarily reducing prediction variance to mitigate overfitting [44]. Boosting iteratively trains homogeneous models by reweighting misclassified samples, progressively minimizing bias [45]. Both use identical base model architectures, making their performance vulnerable to intrinsic algorithmic biases. For example, boosting frequently prioritizes majority class optimization over minority class recognition in class-imbalanced data [46, 47]. Conversely, stacking ensemble learning overcomes these limitations by integrating heterogeneous base models, such as linear regression for additive relationships and gradient-boosted trees for nonlinear interactions, thereby compensating for individual model weaknesses and improve generalizability [48, 49]. This architectural diversity allows stacking to synthesize complementary feature representations, theoretically aligning with Bayesian optimal classifiers, particularly in complex prediction tasks requiring multi-modal pattern recognition.

The training cohort of 1596 samples underwent ten-fold cross-validation, divided into 10 subsets of 159 cases each, optimizing data utility and reducing overfitting [50]. This setup ensured adequate sample size per fold to support feature engineering and hyperparameter tuning for complex algorithms, such as RF and XGB, following the “10 × rule” of 10 samples per feature. With 45 features analyzed, the training set far exceeded this threshold. An independent validation set (*n* = 716) was used for evaluation. There were significant inter-center differences (*P* < 0.05) in clinical parameters such as length of stay, Plt, FIB, fasting blood glucose, DBIL, Caprini score, ASA classification, SSI, and postoperative bleeding. These differences assessed the model’s cross-institutional generalizability. The validation set included 85 VTE positive events, meeting the recommended > 50 positive cases for stable performance estimation (sensitivity/specificity error < 5%), ensuring accurate diagnostic evaluation.

The stacking ensemble model excels in addressing the complex heterogeneity of CRC patients, providing more precise VTE risk stratification than traditional tools. By integrating tumor-specific predictors (e.g., anatomical location, cTNM stage, histological differentiation) with surgical parameters and inflammatory-nutritional biomarkers, the model effectively quantifies the synergistic thrombotic interactions among multimodal risk factors. This allows for the targeted identification of high-risk subgroups requiring intensified prophylaxis. For example, patients with poorly differentiated cT3N1M0 rectal adenocarcinoma undergoing open resection, with Caprini scores ≥ 5 and elevated D-dimer levels (> 2000 ng/mL), may benefit from extended low-molecular-weight heparin regimens, supplemented by serial coagulation profiling. Conversely, the model identifies low-risk groups suitable for anticoagulation de-escalation, such as those with moderately differentiated cT2N0M0 colon carcinoma treated laparoscopically, with Caprini scores < 3 and D-dimer levels < 500 ng/mL, thereby minimizing iatrogenic bleeding risks. SHAP achieves clinically actionable interpretability by visually ranking feature contributions, including surgical site, Caprini score, D-dimer, operation, and length of stay, providing clinicians with an auditable framework for personalizing VTE prophylaxis in accordance with precision oncology paradigms [5, 51].

Among the 22 risk factors studied by Agharezaei et al. [52], bed rest time, advanced age, swelling of both legs, and a history of previous major surgery were the most significant. A previous study investigated the mechanisms of VTE formation and associated risk factors, indicating that blood stasis, abnormal fibrin, and coagulation factors were the leading causes of thrombotic events [53]. VTE pathogenesis in CRC patients results from complex interactions between tumor biology and surgical procedures. Malignant cells release procoagulant substances, such as tissue factor and inflammatory cytokines (IL-6, TNF-α), which activate extrinsic coagulation cascades through platelet activation and thrombin generation [54, 55]. Surgical trauma exacerbates this through three mechanisms: (1) direct disruption of vascular walls exposes subcutaneous collagen, activating platelets and coagulation factor XII; (2) prolonged hospitalization result in immobilization-related venous stasis, particularly in advanced CRC patients with significant tumor burden or central venous catheters, significantly increasing VTE risk compared to early-stage cases [56]; (3) anatomical variations between colorectal subsites—rectal surgeries have a higher VTE incidence than colon surgeries owing to the pelvic venous plexus’s susceptibility to surgical manipulation and lithotomy positioning-induced lower extremity venous compression, while colon surgeries in the supine position minimally affect lower extremity hemodynamics [14, 55, 57]. Open surgeries activate more pronounced inflammatory responses and reduced mobility compared to laparoscopic procedures, resulting in distinct VTE risk profiles. The hypercoagulable state following open surgery is characterized by elevated Caprini scores (≥ 5) and consistently high D-dimer levels (> 2000 ng/mL beyond postoperative day 5), indicating ineffective fibrinolysis and ongoing thrombus formation. In contrast, transient D-dimer increases (< 500 ng/mL within 48 h) are indicative of surgical stress rather than true thrombotic risk. The Caprini score measures Virchow’s triad (stasis, endothelial injury, and hypercoagulability), with higher scores indicating the interaction and cumulative effect of these risk factors [54, 58].

Studies have shown that VTE is associated with poor prognosis in CRC patients [59, 60]. However, some patients requiring thromboprophylaxis may not receive adequate care [16, 50]. Yao et al. [61] conducted a single-center retrospective study on 541 postoperative CRC patients to develop the Sir-Run-Run-Shaw VTE RAM. A multivariable analysis revealed four significant predictors: age ≥ 69 year (*P* < 0.01), preoperative D-dimer ≥ 0.49 mg/L (*P* = 0.004), stage IV malignancy (*P* = 0.018), and perioperative blood transfusion (*P* = 0.004). This model uses a 4-point scoring system (0, 1, ≥ 2) for risk stratification, showing superior discriminative capacity over the Caprini RAM with an AUC of 0.769 *vs* 0.656 [61]. In a separate multicenter prospective cohort study (*n* = 1836), researchers developed the CRC-VTE prediction model using LG. Seven clinical variables were identified as independent risk factors: age ≥ 70 year, lower extremity varicose veins, cardiac insufficiency, female gender, preoperative bowel obstruction, bloody/tarry stool, and anesthesia duration ≥ 180 min. Risk stratification was categorized as low, moderate, high, and very high risk. External validation across regional cohorts confirmed that the model outperformed the Caprini RAM (AUC = 0.72 *vs* 0.59) [5]. While these RAMs provide clinical interpretability and implementation feasibility, Limitations remain. Notably, critical risk factors validated in prior studies are underrepresented in feature selection. Furthermore, conventional LG models frequently fail to capture the complexity of real-world features, as indicated by suboptimal discriminative performance, with AUC values rarely exceeding 0.8. To overcome these limitations, ML methods using electronic health record (EHR) data have gained traction. Agharezaei et al. [52] introduced artificial neural networks to predict VTE using PE and DVT endpoints, achieving 93.23% accuracy in a small cohort (*n* = 294). In contrast, retrospective analysis of 376 hospitalized Chinese patients compared the predictive performance of an ML model to a conventional LG model by Wang et al. [62]. Surprisingly, the ML model did not outperform the linear model on key metrics. Moreover, its clinical utility was limited by sensitivity (68% *vs* 82%), indicating restricted reliability in identifying true-positive VTE cases. A recent study compared three ML models—LG, SVM, and RF—to the traditional Khorana score. The findings demonstrated that these ML models outperformed the Khorana score, regardless of whether they were trained on the full feature set or the same features (AUC = 0.778 ± 0.006) [63]. This was supported further by Qin et al.’s [64] retrospective analysis (*n* = 1191), which compared six ML algorithms (LG, RF, XGB, SVM, MLP, and LSTM) to the Caprini and Khorana scores. The XGB model showed exceptional discriminative ability (training AUC = 0.990; validation AUC = 0.908), and all ML algorithms achieved AUCs greater than 0.8, outperforming traditional scores (Caprini AUC = 0.769; Khorana AUC = 0.646) [64]. However, the study’s single-center design and lack of external validation limit the models’ generalizability.

To address the limitations of studies’ models, we conducted the first attempt to apply ML-stacking in the prediction of VTE complications following CRC surgery. Multicenter data were collected from 1596 and 716 cases. The final prediction model, the stacking model, performed well even in the external validation dataset (training AUC = 0.972; validation AUC = 0.840). The stacking model effectively identifies groups misclassified as ‘low-risk’ by the Caprini score, using early warning signals from multiple base models. In addition, ML models are frequently referred to as “black box” models because they do not explain the underlying logical architecture [65, 66]. To solve this problem, we used feature importance analysis and SHAP value visualization to determine the impact of each feature in the model on the output results. SHAP values allow for the analysis of the stacking decision path, resulting in a “list of high-risk factors” while meeting the dual requirements of interpretability and methodological rigor. The stacking ensemble outperforms the Caprini RAM and individual ML models in predictive performance, clinical applicability, and technical foresight by leveraging heterogeneous model collaboration, dynamic data adaptation, and precise risk assessment. These advancements establish the stacking method as a transformative tool for personalized VTE prophylaxis and management, bridging the gap between population-based guidelines and patient-specific care. In addition to analytical advantages, the stacking model provides operational benefits for clinical implementation. It integrates with hospital information system (HIS) to automate data extraction and risk alerts, thereby reducing manual workloads and minimizing subjective errors. In addition, the model adapts to diverse patient populations and clinical settings through transfer learning and iterative optimization with external datasets.

When transforming the prediction model based on clinical routine indicators into clinical practice, the healthcare system’s implementation strategy must consider technical feasibility, clinical workflow adaptability, and policy compliance. A hierarchical implementation framework should be developed to allow scenario-specific integration, progressing from auxiliary screening to precision interventions. For inpatients undergoing VTE risk assessment, integration begins with embedding the model into national healthcare guidelines, such as those issued by the National Health Commission. This authorization improves interoperability with EHR systems by allowing automated extraction of laboratory data, such as coagulation profiles and nutritional biomarkers, to generate real-time risk scores. These scores improve preoperative screenings, optimize surgical timing, and customize perioperative Management plans. Further integration into nursing monitoring systems allows for threshold-triggered automated alerts. When risk thresholds are exceeded, the system issues notifications instructing clinicians to re-evaluate postoperative patients using lower extremity ultrasonography, repeat coagulation testing, and initiate evidence-based anticoagulation therapy. Simultaneously, healthcare payors, such as medical insurance bureaus, could incorporate these predictive alerts into quality monitoring metrics for DRG payment systems. To Maintain consistent model performance, ML algorithms require dynamic calibration. A performance monitoring module within HIS can track real-time discrepancies between predicted and actual outcomes, initiating automatic model retraining if the AUC decreases by more than 5%. Privacy-preserving computational strategies, such as federated learning architectures, enable secure model training across multiple institutions using encrypted routine test data, protecting patient privacy. As clinical implementation progresses, the model transitions from generating passive alerts to actively guiding advanced decision-making, eventually informing personalized therapeutic strategies.

Our study has addressed several critical limitations from previous studies, but further research is needed to fully resolve these challenges. Some patients were excluded from the analysis owing to clinical data and perioperative treatment factors, potentially introducing bias. Because of limited data access, the study did not incorporate imaging biomarkers such as upper abdominal CT findings or molecular profiling data, including immunohistochemical markers and genetic typing. Relying solely on clinical parameters may overlook significant predictors such as EGFR mutation status and PD-L1 expression levels, compromising the model’s ability to address diverse patient populations and limiting clinical decision support systems. Expanding sample sizes through prospective cohort validation and multicenter randomized controlled trials is critical for demonstrating the model’s clinical efficacy. A pragmatic method could include hybrid frameworks that integrate ML with established risk assessment tools, such as using ML algorithms to dynamically update Caprini scores based on real-world data. In addition, the impact of perioperative interventions on clinical outcomes requires systematic evaluation, particularly in their interactions with AI-driven predictive models. These multidimensional enhancements will address existing gaps in model generalizability and therapeutic relevance, advancing toward clinically actionable AI solutions.

## Conclusions

In this study, ML algorithms were used to develop models for predicting VTE in CRC patients undergoing radical resection. In both external validation and inter-model comparisons, the stacking model demonstrated superior predictive performance. Integrating this model into electronic medical records is recommended to assist clinicians in identifying high-risk patients and developing personalized prevention strategies. Future studies should aim to increase the multicenter sample size and conduct prospective validation studies.

## Data Availability

The datasets used and/or analysed during the current study are available from the corresponding author on reasonable request.

## Abbreviations

ML: Machine learning
VTE: Venous thromboembolism
CRC: Colorectal cancer
DVT: Deep vein thrombosis
PE: Pulmonary embolism
AI: Artificial intelligence
CT: Computed tomography
LG: Logistic regression
RF: Random forest
XGB: EXtreme gradient boosting
SVM: Support vector machine
MLP: Multilayer perceptron
SHAP: Shapley additive explanation
ROC: Receiver operating characteristic
RAM: Risk assessment model
AUC: Area under the curve
WBC: White blood cell
Hb: Hemoglobin
APTT: Activated partial thromboplastin time
ALT: Alanine aminotransferase
ASA: American Society of Anesthesiologists
Mb: Myoglobin
AT-III: Antithrombin III
PA: Prealbumin
BMI: Body mass index
Plt: Platelet count
PT: Prothrombin time
AST: Aspartate aminotransferase
FIB: Fibrinogen
Cr: Serum creatinine
UA: Uric acid
TG: Triglyceride
TC: Total cholesterol
LDL: Low-density lipoprotein
FBG: Fasting blood glucose value
CKMB: Creatine kinase isoenzymes
DBIL: Direct bilirubin
IBIL: Indirect bilirubin
DM: Diabetes mellitus
HP: High blood pressure
CHD: Coronary heart disease
ICH: Intracerebral hemorrhage
CVT: Cerebral thrombus
PUI: Preoperative pneumonia
po-CVT: Postoperative-CVT
POP: Postoperative pneumonia
AL: Anastomotic leak
SSI: Surgical site infection
IAI: Intra-abdominal infection
Po-bleeding: Postoperative-bleeding
EHR: Electronic health record
HIS: Hospital information system
